# Adoption and use of the 7-1-7 target for timely outbreak detection, notification and early response in the human and animal sectors in humanitarian crisis-affected South Sudan: A mixed-methods study during 2025

**DOI:** 10.12688/wellcomeopenres.25112.1

**Published:** 2026-02-16

**Authors:** Joseph Daniel Wani Lako, Angelo Goup Thon, Agol Malak Kwai, Santino Ngong Garang, John Mabior Aguto, Santino Makuach, Santo Malek Deng, Harriet Akello Pasquale

**Affiliations:** 1 Department of Biotechnology, University of Juba, Juba, Central Equatoria, South Sudan; 2 Department of Health Security, SURGE, Capacity Development, and One Health, Ministry of Health, Juba, South Sudan; 3 Directorate of Veterinary Services, Ministry of Livestock and Fisheries, Juba, South Sudan; 4 Directorate of International Health Coordination (IHC), Ministry of Health, Juba, South Sudan

**Keywords:** pandemic preparedness, disease outbreaks, early diagnosis, disease notification, response, bottleneck, vulnerable populations, resource-limited settings

## Abstract

**Background:**

Globally, the 7-1-7 target (detect within 7 days, notify within 1 day, respond within 7 days) was introduced to improve outbreak response. South Sudan adopted the 7-1-7 framework in August 2023. This study aimed to describe the process and challenges of adopting the 7-1-7 framework, assess the performance of outbreaks in the animal and human sector against the 7-1-7 target, and explore the challenges for 7-1-7 adoption in humanitarian settings.

**Methods:**

This mixed-methods study comprised a checklist-based assessment of 7-1-7 adoption at national and subnational levels, extraction of dates and bottlenecks from the 7-1-7 consolidated spreadsheet for outbreaks that occurred before (2013-July 2023) and after (August 2023-July 2025) 7-1-7 adoption, and in-depth interviews and focus group discussions among key stakeholders to explore the challenges in 7-1-7 adoption.

**Results:**

7-1-7 was successfully adopted at the national level and in four of ten states and three administrative areas, facilitated by strong leadership, and project funding and technical support from the 7-1-7 Alliance. Due to lack of funds, 7-1-7 was not adopted nation-wide. Data for applying the 7-1-7 framework was available in 9/25 (36%) and 9/9 (100%) outbreaks that occurred before and after adoption, respectively. None of the five outbreaks from the animal sector had complete data for assessing the target. Of 18 outbreaks assessed, 44% met all targets with 50%, 91% and 56% achieving detection, notification and response targets, respectively. Delayed health seeking, poor internet connectivity, and inadequate resources were bottlenecks for timely detection, notification, and response, respectively. Non-functional health facilities and competing priorities were major barriers to adopting the 7-1-7 framework in humanitarian settings.

**Conclusion:**

South Sudan successfully adopted 7-1-7 at the national-level. Sustainable funding is required to expand 7-1-7 to all states, including humanitarian settings, establish a surveillance information system for the animal sector and improve timely outbreak response.

## Plain Language Summary

To speed-up detection and response to public health threats, South Sudan adopted the 7-1-7 framework in 2023 to achieve detection within 7 days, notification within 1 day, and complete early response by 7 days. This study examined how well the framework has been adopted, and how effectively outbreaks that occurred in human and animal sectors between 2013 and 2025 met the 7-1-7 target.

In 2025, we used checklists to assess 7-1-7 adoption at national and subnational levels, extracted key dates and bottlenecks for outbreaks that occurred before (2013-July 2023) and after (August 2023-July 2025) 7-1-7 adoption, and conducted discussions with key stakeholders to explore the challenges in 7-1-7 adoption.

7-1-7 was successfully adopted at the national level and in four of ten states and three administrative areas, facilitated by strong leadership. Due to lack of funds, 7-1-7 was not adopted nation-wide. Data availability for applying the 7-1-7 framework to outbreaks improved after adoption of the metrics- from 36% before to 100% after adoption.

Of 18 outbreaks assessed, 44% met all targets with 50%, 91% and 56% achieving detection, notification and response targets, respectively. Delayed health seeking, poor internet connectivity, and inadequate resources were bottlenecks for achieving detection, notification, and response targets, respectively. None of the five outbreaks from the animal sector had complete data and there was no functioning surveillance system for animal outbreaks. Non-functional health facilities, displacement, damaged infrastructure, and competing interests were major barriers to adopting the 7-1-7 framework in humanitarian settings.

The limitations of this study were the small number of events assessed and inability to determine the sustainability of the framework’s adoption. To scale up the framework across the entire country and ensure long-term viability, increased and sustained funding is needed. Addressing the bottlenecks that delay outbreak detection, notification, and response would improve outbreak preparedness.

## Introduction

The ability of a country's health system to detect and respond promptly and effectively to public health events of concern determines whether the event can be brought under control or whether it will eventually escalate into an outbreak. Existing deficiencies within health systems in detecting, notifying, and responding to public health events in a timely manner contributed to the recent COVID-19 pandemic and the ongoing outbreaks of mpox in the African region. Thus, there is a need for timeliness metrics for detection, notification, and response, to help improve the speed of these processes.

In July 2021, Frieden *et al.*, from Resolve to Save Lives (RTSL) proposed a new global timeliness metrics of 7-1-7 to improve early detection and rapid control of public health events arising from suspected infectious disease outbreaks ^1^. The metric is designed to work as follows: every suspected infectious disease outbreak is detected within 7 days of emergence (first-7); the outbreak is notified to the appropriate public health authorities within 1 day of detection (next-1); and effective early response measures with objective benchmarks are put in place within 7 days of reporting (second-7). Clear definitions and milestones for emergence, detection, notification, and completion of early response actions enable health systems in countries to assess their own performance against the 7-1-7 metrics and identify the enablers and barriers to achieving this target ^2^.

In 2022, a study conducted in five countries found that it is feasible to apply the 7-1-7 metrics to public health events that had occurred in the past. Of the 41 events assessed, the first-7 target was met in 54%, the next-1 target was met in 71%, and the second-7 target was met in 49% ^2^. Altogether, 27% of events met the complete 7-1-7 target, with bottlenecks and enablers identified for each of the components ^2^. However, a substantial proportion of countries have been unable to adopt and use this timeliness metrics because they lack systems to regularly collect and report on such timeliness data ^3^.

The Health Security Preparedness Division of the World Health Organization (WHO), acknowledging the utility of the 7-1-7 metrics in outbreak response, recommended generating evidence on how the 7-1-7 metrics has been adopted and used in the twenty countries that have adopted it ^4^. In light of this, the 7-1-7 Alliance issued a call in 2024 for proposals from front-runner countries to use operational research to generate evidence on the adoption and use of the 7-1-7 target for performance improvement ^5^.

 South Sudan, the world’s youngest nation, was one of the front-runner countries to adopt the 7-1-7 metrics in August 2023 by training key stakeholders and integrating the metrics into the existing infectious disease surveillance systems of human, animal, and environmental health sectors. By 2024–2025, 7-1-7 was expanded to the subnational level, and key stakeholders from four selected states were trained. 7-1-7 was adopted in South Sudan despite the country facing complex public health challenges due to recurrent humanitarian crises, fragile health systems, and frequent disease outbreaks such as cholera, measles, malaria, meningitis, zoonotic diseases and viral hemorrhagic fevers. In addition, recurrent flooding, population displacement, and influx of refugees further compound the risk of delayed detection, notification, and inadequate responses to outbreaks. 

South Sudan has a unique experience of adopting and using the 7-1-7 metrics within a complex public health system threatened by humanitarian crises. Thus, South Sudan, with financial support from the 7-1-7 Alliance, aimed to conduct operational research to generate comprehensive evidence on the adoption and use of the 7-1-7 metrics. Such a comprehensive assessment required a mixed-methods design with both quantitative and qualitative approaches to understand the extent of adoption, its enablers and challenges, and the use of the 7-1-7 metrics. The evidence from the study would not only help to optimize the implementation of the 7-1-7 metrics in the country but would also provide the knowledge and practical guidance for adopting and using the metrics in other countries dealing with humanitarian crises.

In this regard, this operational research was conducted with three objectives; *Objective 1*: to describe the adoption process and challenges in adopting the 7-1-7 framework at the national level and in four states of South Sudan; *Objective 2*: among all the outbreaks that occurred in South Sudan from January 2013 to July 2025, to assess the proportion of outbreaks in which the 7-1-7 framework could have been applied, and to describe the overall performance of the timeliness metrics, along with the enablers and bottlenecks in achieving the target; *Objective 3*: to explore the challenges in expanding the 7-1-7 metrics to humanitarian settings in South Sudan.

## Methods

### Study design

***Objective 1:*** This was an explanatory mixed-methods (sequential: QUAN-QUAL) study. The quantitative component was a cross-sectional study with primary data collection using the adoption milestone checklists (Annex 1, Extended data) ^6^. The qualitative component was a descriptive study using in-depth interviews as the method of data collection.

***Objective 2:*** This was an explanatory mixed-methods (sequential: QUAN-QUAL) study. The quantitative component was a cohort study using secondary data routinely collected by the national surveillance unit to assess the 7-1-7 metrics using the consolidated spreadsheet (Annex 2, Extended data) ^6^. The qualitative component was a descriptive study using in-depth interviews as the method of data collection.

***Objective 3:*** This was a descriptive qualitative study using in-depth interviews and focus group discussions as methods of data collection.

### Study setting

***General***

The Republic of South Sudan is a landlocked country in the Eastern African region, with an estimated population of 12.2 million in 2025 ^7^. South Sudan is administratively divided into states (10), counties (80), and payams (509), with the lowest administrative level being the boma (2,500). The country has a nominal gross domestic product of USD 341 per capita.

South Sudan, the world’s youngest nation, gained independence from Sudan on July 9, 2011, after decades of civil war. Post-independence, there has been a high influx of refugees and returnees from Sudan (because of the Sudan Crisis), primarily sheltered in camps around the borders with limited access to basic amenities. The country has a diverse population with over 60 ethnic groups, and experiences internal conflicts due to widespread poverty, political instability, ethnic tensions, and food insecurity. More than 80% of the population lives in rural areas and has poor access to safe water, improved sanitation, poor road infrastructure and electricity. Flooding is common in the catchment area of the River Nile during the rainy season. Thus, the country remains in a constant humanitarian crisis.

***Specific setting***

**Organization of the health system in the county**

South Sudan has a three-tiered health system, which is challenged by limited infrastructure, widespread poverty, political instability, an inadequate healthcare workforce and low government funding. Health service delivery is largely dependent on funding from development partners for medicines, medical supplies, and salaries for health workers. The Ministry of Health (MoH) is responsible for policymaking, planning, regulating, and coordinating health services. Services are delivered primarily through public sector facilities and supported by non-governmental organizations (NGOs), international agencies, and private providers. Especially in humanitarian crises settings like refugee and internally displaced persons’ camps, and flooded areas, services are mainly delivered by NGOs in collaboration with the MoH and international agencies.

**Laboratory services**

At the county level, there are Level-I laboratories that provide basic investigations using rapid testing kits for diseases like malaria, typhoid, and HIV/AIDS. At the state level, there are Level-II laboratories that provide microscopy and PCR-based tests for the detection of diseases like tuberculosis, measles, and COVID-19. At the national level, there is a Level-III laboratory with infrastructure for conducting culture and drug susceptibility tests. In case of suspected outbreaks, the samples are shipped to the Level-III National Public Health Laboratory in Juba. Samples collected routinely are transported directly from health facilities or the community to the county level, utilizing all available local means of transportation. From the county level, samples are transported by road or via UN Humanitarian Air Service flights to Juba. Partners and UN agencies are responsible for facilitating sample delivery to Juba’s National Public Health Laboratory when needed. The majority of investigations are provided free of cost or for minimal user fees in public health laboratories.

**Health management and information systems (HMIS)**

Efforts have been made to institutionalize the District Health Information System (DHIS 2.0) and Early Warning, Alert and Response System (EWARS) at the primary care facilities. However, due to limited infrastructure and interruptions in the internet, data entry at these facilities is limited. The HMIS assessment of South Sudan (2018) showed that adequate HMIS infrastructure was available only in 43% of the facilities. Lower-level health facilities share data with county surveillance offices, where it is entered into DHIS 2.0 or EWARS. This process compromises the overall completeness and timeliness of reporting. In addition, some disease-specific programs, including those for Neglected Tropical Diseases and HIV, still run with parallel/vertical reporting systems, resulting in fragmentation of the health information system.

***Adopting, implementing and evaluating the 7-1-7 metrics for the current project***

**Detection of the outbreak (First-7)**

Infectious disease surveillance in South Sudan is mostly passive, but active surveillance occurs once a priority public health event is detected ^8^. Staff in health facilities evaluate the index patient using standard case reporting forms and case investigation forms. The patient's community is then visited and an active search for cases is conducted to confirm the presence of an outbreak. Additionally, as part of case investigation, appropriate specimens are collected and sent to public health laboratories for testing.

There is also a hotline number (6666) for the general public to report any priority public health disease outbreak occurring in their community, and these reports are included in the reported public health events records.

County surveillance officers and health facility staff are primarily involved in the verification activities. They document all the details of outbreak investigations in the paper-based facility register form. The date of emergence of the event is ascertained in line with the 7-1-7 framework definition. The date of detection of an outbreak is decided based on standard definitions which take into account the endemicity and endemic threshold of the disease. For endemic diseases, any increase in the number of suspected cases over and above the defined endemic threshold in bomas or counties is considered as an outbreak. For non-endemic diseases, even a single confirmed case is considered as an outbreak.

In South Sudan, where livestock play a critical role in livelihood and food security, detection and response systems in the animal health sector are established to manage zoonotic diseases (e.g., Rift Valley fever, anthrax, brucellosis). Veterinary officers and Community Animal Health Workers (CAHWs) recognize the signs of disease outbreaks in livestock. Samples are collected and transported to the national veterinary laboratories to confirm the diagnosis.

**Notification of the outbreak or public health event (next-1)**

When an outbreak is detected, the health facility in charge enters its details into EWARS or DHIS 2.0, from where it is transmitted to the county, state, and national surveillance units and rapid response teams. In health facilities where the infrastructure for entering data into online platforms is unavailable, the alert is reported to the county surveillance unit through phone calls or by sending the paper-based facility register form. The public health event is then entered into EWARS or DHIS 2.0 from the county or state hospitals.

In the animal sector, direct channels for notification are established between CAHWs and veterinary authorities through real-time notification systems, using mobile apps and radio communication.

**Early Response Actions (second-7)**

The Department of Health Security, Emergency Workforce Development and One Health oversees all the response activities after an outbreak is notified. Integrated Disease Surveillance and Response (IDSR) teams, personnel of Public Health Emergency Operations Centres (PHEOC), and national rapid response teams (RRT) are involved in executing the following response actions:

- Component 1: initiate investigations and deploy investigating team
- Component 2: conduct epidemiological analysis
- Component 3: obtain laboratory confirmation of the outbreak
- Component 4: initiate appropriate management and IPC in health facilities
- Component 5: initiate appropriate public health counter measures
- Component 6: initiate communication activities
- Component 7: establish a coordination mechanism

Daily reports are submitted by personnel from IDSR, PHEOC and RRT via email to the Department of Health Security, highlighting the progress of response activities. A final report of all the activities and achievements is also prepared and submitted at the end of the outbreak response. 

**7-1-7 assessment**

National surveillance officers assess the date of emergence, detection and notification as soon as an outbreak is notified through EWARS or DHIS 2.0. They also request a copy of the paper-based facility register form. State and county surveillance officers collect further details to assess the first two components (first 7 and next 1) of the 7-1-7 framework, identify their bottlenecks, and report these to the national surveillance officers.

The national surveillance officers closely examine the daily reports shared by the personnel from IDSR, PHEOC, and RRTs. Concurrently, during daily discussions with IDSR, PHEOC, and RRTs, surveillance officers identify the bottlenecks and enablers for the timely implementation of response measures.

All assessment details are documented in the 7-1-7 data consolidation sheet, and the information is presented to Incident Managers and national steering committees.

### Study population

***Objective 1***

**Quantitative Component**

A checklist-based assessment was conducted at national level and in the four states where the 7-1-7 metrics have been adopted.

**Qualitative Component**

All individuals involved in the adoption of the 7-1-7 framework at the national and state levels were eligible for the study. Criterion-based purposive sampling was used to select participants to understand the adoption process, its facilitators and challenges. In total, six interviews were conducted.

***Objective 2***

**Quantitative Component**

The public health events that occurred during January 2013 to July 2025 and assessed using 7-1-7 framework and recorded in the 7-1-7 Data Consolidation Spreadsheet were included in the study.

**Qualitative Component**

All individuals involved in assessing events using the 7-1-7 framework and those involved directly in detecting and responding to the public health events were eligible. In total, nine interviews were conducted among the surveillance officers, rapid response team members, and other healthcare providers. The number of in-depth interviews was guided by the saturation of information, which was assessed after five in-depth interviews.

***Objective 3***

All individuals involved in the adoption of the 7-1-7 framework and tackling the humanitarian crises at the national, state, and county levels were eligible for the study. Criterion-based purposive sampling based on participants’ experience with different humanitarian crises was used to select participants. In total, six in-depth interviews, and one focus group discussion among community volunteers affected by the humanitarian crisis were conducted.

### Data sources, data variables and data collection

***Quantitative component***

**Objective 1**

In June 2025, a trained surveillance officer filled the adoption checklist to assess the 7-1-7 adoption process and the structures in-place at the national level and in the four states, in consultation with the national, and state surveillance officers. The data from the paper-based checklist was entered into Microsoft Excel for analysis.

**Objective 2**

All events reported between January 2013 and July 2025 were extracted from the EWARS system. Surveillance officers accessed the 7-1-7 Data Consolidation Spreadsheet (Annex 2, Extended data) and included all events present in the database for analysis ^6^. All relevant dates (date of emergence, date of detection, date of notification, date of early response initiation, date of early response completion, and date of completion of each of the seven response components) were extracted for analysis. Additionally, identified bottlenecks and enablers for detection, notification and response within 7-1-7 timelines were extracted for analysis.

***Qualitative Component***

A trained surveillance officer fluent in the local language and trained in qualitative research conducted all in-depth interviews and the focus group discussion. Participants were approached in-person and informed consent was taken. The interviews and discussion were conducted at a place and time convenient to the participants, ensuring privacy. An iterative approach was used, and the interviews were conducted until the point of information saturation. Interview guides (Annex 3, 4, and 5, Extended data) were used for conducting interviews/discussions ^6^. The interviews/discussions were audio-recorded. If participants did not consent to audio recording, field notes were used to prepare transcripts. Interviews were conducted in a location convenient to the participant, and each interview lasted for 30 to 45 minutes.

### Data analysis

***Quantitative Component***

**Objective 1**

Data collected on the paper-based checklist were entered and analysed using Microsoft Excel. Adoption milestones completed at the national and state levels were described.

**Objective 2**

Four analyses were conducted: i) Among all the events reported to EWARS during January 2013 to July 2023 (prior to adoption of 7-1-7) and during August 2023 to July 2025 (after adoption), we summarized the number and proportion of verified events denoted as outbreaks, and the number and proportion of outbreaks to which the 7-1-7 metrics could be applied; ii) The overall 7-1-7 target, and the number and proportion of the events achieving: First 7 - detected within seven days of emergence; First 1 - notified within one day of being detected; Second 7 - all seven early response components completed within seven days of notification were calculated. The number and proportion of events for which the 7-1-7 target was fully met within the time frame was also calculated. iii) Achievement of the 7-1-7 target among outbreaks that occurred before and after adoption of 7-1-7 was calculated. iv) Frequencies and percentages were used to summarize enablers and bottlenecks identified for each event. Findings were reported by using ‘Strengthening the Reporting of Observational studies in Epidemiology’ (STROBE) guidelines ^9^.

***Qualitative Component***

Transcripts were prepared in English within one week of each interview/discussion using notes and audio recordings. Two investigators conducted a manual content analysis to identify codes. A third investigator reviewed the analysis, and the decision on coding and theme generation were made in consensus. Similar codes were combined into themes. The codes/themes were related to the original data to ensure that the results reflected the data. The findings were reported by using ‘Consolidated Criteria for Reporting Qualitative Research’ (COREQ) guidelines ^9^.

## Statement on Patient and Public Involvement

Patients and the public were not involved in the planning or conduct of this study.

## Ethical Approval and Consent to Participate

The study was conducted according to the guidelines of the Declaration of Helsinki. Ethical approved was granted by the Ministry of Health, Research Ethics Review Board of Republic of South Sudan (Reference number: MOH/RERB/A/71/11/2024). Written informed consent was obtained from all participants before interviews. No identifying personal data have been reported.

## Results

### Objective 1: Adoption of 7-1-7 framework

The 7-1-7 framework was introduced in South Sudan in 2023. Over the course of that year, different adoption milestones were achieved at the national level, including the identification of a government champion, the formation of a coordination team, the engagement of relevant stakeholders, the training of responsible staff and the piloting of its use. In the latter half of 2024, the framework was introduced in four selected states in a staggered manner, where adoption milestones were also achieved, as detailed in Supplementary Table 1, Annex 6, Extended data ^6^. The 7-1-7 framework has not yet been adopted in the other six states and three administrative areas.

**Figure 1.**
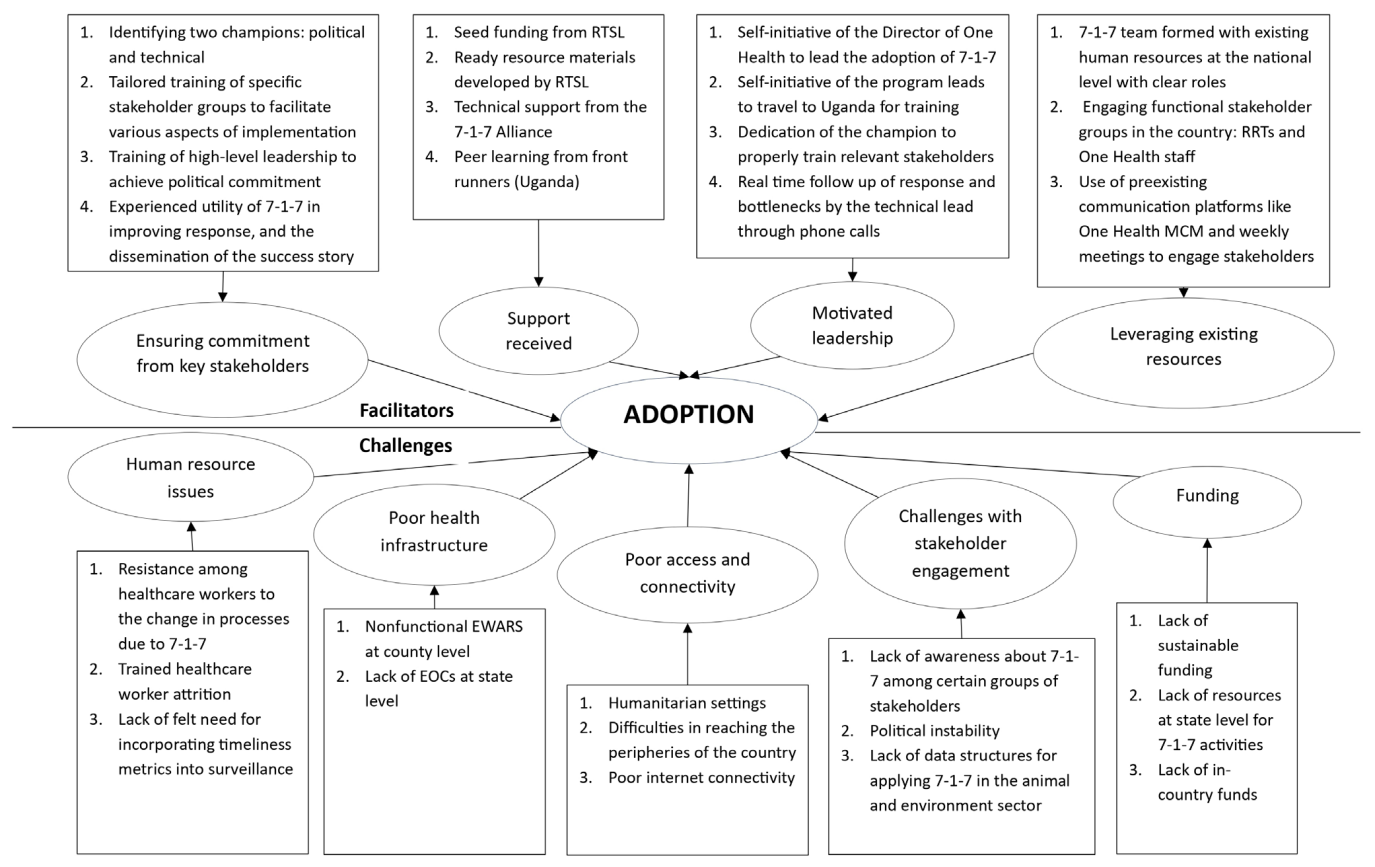
Thematic diagram of the facilitators and challenges in adopting 7-1-7 in South Sudan. Figure 1 depicts the facilitators and challenges associated with adopting the 7-1-7 metrics in South Sudan, as identified through in-depth interviews. PMEP: Programmatic Management for Epidemic Preparedness; RTSL: Resolve to Save Lives; RRTs: Rapid Response Teams; MCMs: Multisectoral Coordination Mechanisms; EWARS: Early Warning, Alert and Response System; EOCs: Emergency Operations Centre

Through qualitative interviews with key stakeholders, several factors were identified that facilitated the adoption of 7-1-7 at the national and state levels ( Figure 1 and Supplementary Table 2, Annex 6, Extended data) ^6^. Personnel from key government ministries (Humanitarian Affairs and Disaster Management, Livestock and Fisheries, Environment and One Health) learned about 7-1-7 at a virtual training session on epidemic preparedness, after which the Director of Health Security, SURGE Capacity Development and One Health assumed responsibility for adopting the timeliness metrics at the national and state level. Tailored training workshops were conducted- one each for high-level leadership, One Health focal points and rapid response teams. The adoption process and trainings were supported by the financial and technical support from RTSL. In the early stages of adoption, the utility of 7-1-7 was evident to all the stakeholders, as it enabled the rapid procurement of yellow fever vaccines for the first time.

Several challenges were encountered during adoption. There was no in-country funding, and states lacked the resources required to function effectively, including computers, vehicles, physical infrastructure, and trained manpower. Healthcare workers did not initially understand the importance of the metrics, as surveillance systems were already in place. The recurring problem of trained government workers vacating their posts due to low salaries and moving to NGOs or other countries was also reported. Poor internet connectivity was a nationwide challenge, as was poor road connectivity to peripheral areas; some areas were reported to be completely cut off because of floods and humanitarian crisis ( Figure 1).

### Objective 2: Use of 7-1-7 frame work

**Figure 2.**
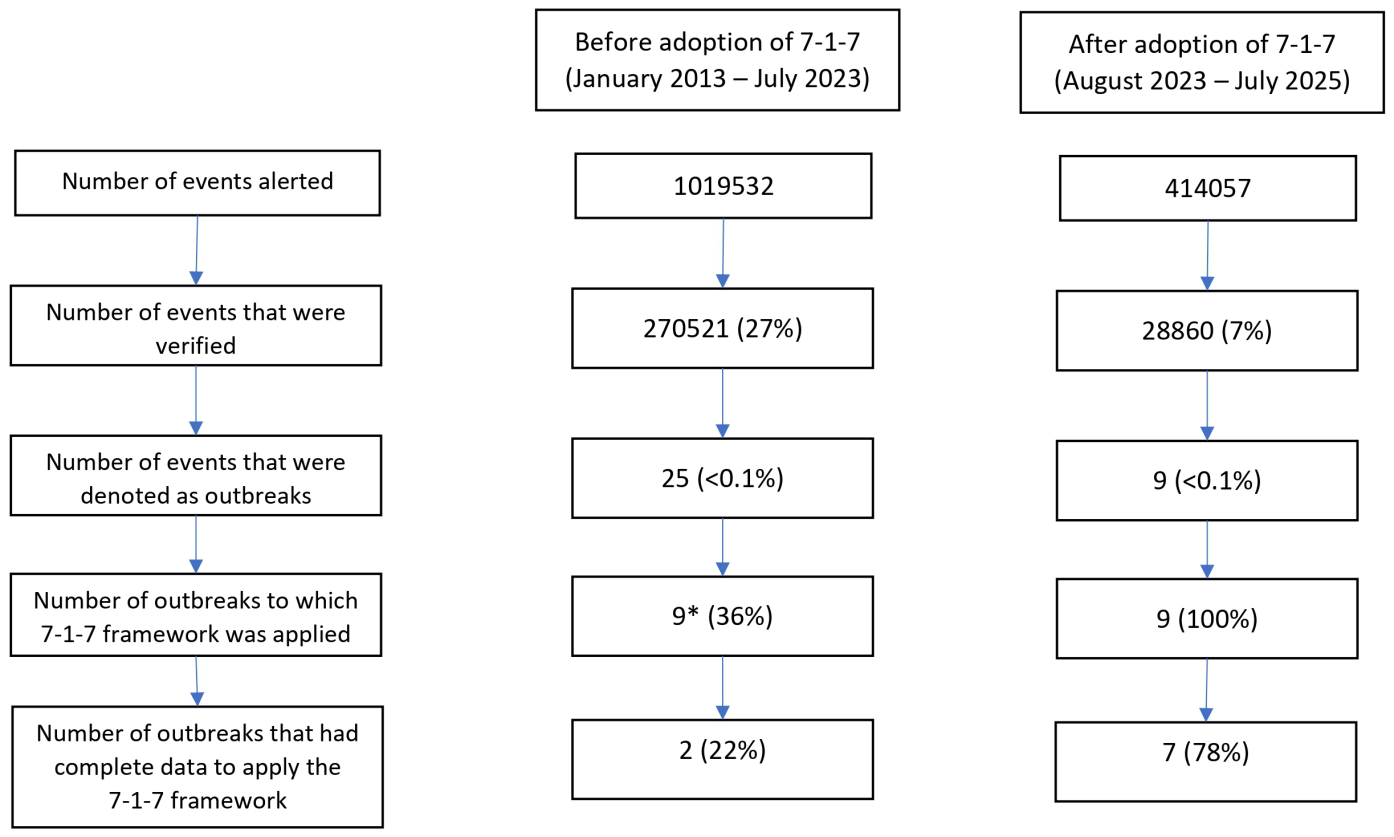
Number of events alerted, verified, denoted as outbreaks and applied 7-1-7 framework in South Sudan. *Any data was available for only 9 out of 25 outbreaks

From the adoption of the 7-1-7 framework in August 2023, the timeliness metrics have been applied retrospectively to verified outbreaks from 2013 onwards, for which data were available. Between January 2013 and July 2023, more than 1 million events were alerted, out of which 27% were verified. These events encompassed human infectious diseases, zoonotic diseases, chemical events, events due to pollution, fatal accidents, etc. Eventually, only 25 events were denoted as outbreaks, and the 7-1-7 framework was applied to nine (36%) of them, of which only two (22%) had complete data (dates of emergence, detection, notification and response) recorded. After August 2023, of 414,057 events alerted, only 7% were verified and nine were confirmed as outbreaks. In contrast to the pre-adoption period, the 7-1-7 framework was applied to all the nine outbreaks, and seven (78%) had complete data for applying the framework ( Figure 2).

Before the adoption of 7-1-7, dates of detection, notification, and response were available in only 78%, 33%, and 22% of outbreaks to which the framework was applied, respectively. However, after adoption, missing data decreased, with dates of notification missing in only 11% and dates of response missing in 22% of outbreaks. For outbreaks among animals, dates of detection were available in four out of five cases, but dates of notification were available in only one case and no dates of response were recorded. The human sector did comparatively better, with 92%, 77% and 69% of data availability, respectively ( Table 1).

**Table 1.** Timeliness of detection, notification and response of public health events against the 7-1-7 target (N=18)

Public Health Event	Total number of public health events for which 7-1-7 metrics was applied	Number of Public Health Events with Date of Emergence and Detection Recorded, n(%)**	Detection (target=7 days)		Number of Public Health Events with Date of Detection and Notification Recorded, n(%) **	Notification (target=1 day)		Number of Public Health Events with Date of Notification and Response Recorded, n(%) **	Completed Early Response (target = 7 days)		Number of Public Health Events with All Dates Recorded, n(%) **	717 met target (all targets met), n(%) ^&^
			Median (IQR) days	Met target, n(%) #		Median (IQR) days	Met target, n(%) $		Median (IQR) days	Met target, n(%) ^		
**All**	18	16 (89)	8 (2–19)	8 (50)	11 (61)	0 (0–0)	10 (91)	9 (50)	7 (2–10)	5 (56)	9 (50)	4 (44)
**Before** *	9	7 (78)	15 (10–31)	2 (29)	3 (33)	0 (0–0)	3 (100)	2 (22)	26	1 (50)	2 (22)	1 (50)
**After** ^†^	9	9 (100)	6 (2–8)	6 (67)	8 (89)	0 (0–0)	7 (88)	7 (78)	7 (2–10)	4 (57)	7 (78)	3 (43)
**Animals**	5	4 (80)	22 (13–30)	1 (25)	1 (20)	0	1 (100)	0 (0)	-	-	0 (0)	-
**Humans**	13	12 (92)	7 (2–14)	7 (58)	10 (77)	0 (0–0)	9 (90)	9 (69)	7 (2–10)	5 (56)	9 (69)	4 (44)

*Before the adoption of 7-1-7: 2013 to July 2023; ^†^After the adoption of 7-1-7: August 2023 to July 2025; **Percentage calculated with total number of events as the denominator; # Percentage calculated with Number of Public Health Events with Date of Emergence and Detection Recorded as the denominator; $ Percentage calculated with Number of Public Health Events with Date of Detection and Notification Recorded as the denominator; ^Percentage calculated with Number of Public Health Events with Date of Notification and Response Recorded as the denominator; & Percentage calculated with Number of Public Health Events with All Dates Recorded as the denominator; 0 days indicates less than 24 hours.

The 7-1-7 timeliness metrics were applied to 18 outbreaks between 2013 and July 2025. Five outbreaks occurred in animals (cattle anthrax, fowl pox, rabies in dogs, East Coast fever, and foot and mouth disease in cattle); while the remainder affected humans (yellow fever, tungiasis, hepatitis E (2), Covid-19, malaria, type 2 polio, meningitis (2), measles, cholera, monkey pox, and rabies). Among these outbreaks, 50% were detected within seven days, with a median (IQR) time to detection of 8 (2–19) days. All but one (91%) were notified in zero days (within 24 hours, and 56% had their early response completed within seven days, with a median (IQR) of 7 (2–10) days. Overall, only four events met the overall 7-1-7 target ( Table 1).

Five factors were reported to enable timely detection, six for notification, and four for timely response activities. Well-trained health professionals were reported as an enabler for timely detection of an outbreak in eight out of ten events, for timely notification in six out of nine events, and for timely response in three out of four events ( Table 2).

**Table 2.** Enablers for timely detection, notification and early response of outbreaks in South Sudan 2013–2025 (N=18).

	Themes of enablers	Categories of enablers	Number	(%) *
**Enablers for ** **detection**		**Number of events with reported enablers for ** **detection**	**10**	
	Clinical or Health Care Worker	Health professional well trained in surveillance and response	8	(80)
		Good awareness or clinical suspicion by health workers	5	(50)
		Availability of trained health workers in the community	2	(20)
	Data Systems	Functioning electronic surveillance/reporting systems (e.g., EPR system)	1	(10)
	Planning and Procedures	Increased procedures in place for surveillance due to outbreak in neighbouring country	1	(10)
**Enablers for ** **notification**		**Number of events with reported enablers for ** **notification**	**9**	
	Clinical or Health Care Worker	Health professional well trained in surveillance and response	6	(67)
		Good awareness or clinical suspicion by health workers	2	(22)
	Data Systems	Functioning electronic surveillance/reporting systems (e.g., EWARS and IDSR)	2	(22)
	Resources and Procurement	Presence of resources to notify (Internet connection)	2	(22)
	Planning and procedures	Timely deployment of the National Rapid Response Team	2	(22)
		Adequate procedures in place to notify	1	(11)
**Enablers for ** **response**		**Number of events with reported enablers for ** **response**	**4**	
	Clinical or Health Care Worker	Health professional well trained in surveillance and response	3	(75)
	Resources and Procurement	Adequate availability of countermeasures or personal protective equipment	1	(25)
	Planning and Procedures	Timely deployment of the National Rapid Response Team	1	(25)
	Coordination	Presence of One Health information sharing/ collaboration	1	(25)

EPR: Emergency preparedness and response; EWARS: Early warning and response system; IDSR: Integrated disease surveillance and response; * Percentage calculated with number of events with reported enablers as the denominator

Seven bottlenecks were reported for timely detection, six for timely notification, and fifteen for early response activities. Common bottlenecks for detection included delays in care seeking by patients, delays in specimen transportation, low clinical suspicion among health workers, and their limited knowledge regarding disease surveillance and response. Airtime shortage was reported as a bottleneck for notification in two out of five events, while the most frequently reported bottleneck for timely response was the limited availability of countermeasures and personal protective equipment ( Table 3).

**Table 3.** Bottlenecks for timely detection, notification and early response of outbreaks in South Sudan 2013–2025 (N=18)

	Themes of bottlenecks	Categories of bottlenecks	Number	(%) *
**Bottlenecks for ** **detection**		**Number of events with reported bottlenecks ** **for detection**	6	
	Clinical or Health Care Worker	Health professional with inadequate training in surveillance and response	2	(33)
		Low awareness or clinical suspicion by health workers	2	(33)
	Patient and Community	Delay in care-seeking by patient	2	(33)
	Laboratory	Delayed specimen collection	2	(33)
		Delayed specimen transportation	1	(17)
	Resources and Procurement	Inadequate resources for rapid resource mobilisation (fuel shortage)	1	(17)
		Delayed approvals (e.g. bureaucratic, regulatory)	1	(17)
**Bottlenecks for ** **notification**		**Number of events with reported bottlenecks** ** for notification**	5	
	Resources and Procurement	Inadequate resources to notify (Airtime shortages)	2	(40)
	Data Systems	Technological challenge for electronic surveillance/reporting systems (e.g., network coverage)	1	(20)
	Event Characteristics	Access issues (e.g., remote, fragile, conflict settings, climate conditions)	1	(20)
	Clinical or Health Care Worker	Inadequate clinical surveillance focal point (posts deserted due to not receiving salary)	1	(20)
		Inadequate procedures in place for event notification	1	(20)
	Coordination	Inadequate One Health information sharing/ collaboration	1	(20)
**Bottlenecks for ** **response**		**Number of events with reported bottlenecks** ** for response**	9	
	Resources and Procurement	Limited availability of countermeasures or personal protective equipment	4	(44)
	Event Characteristics	Access issues (e.g., remote, fragile, conflict settings, climate conditions)	2	(22)
	Planning and Procedures	Inadequate risk assessments, preparedness, or response plans	2	(22)
		Inadequate resources for response initiation or rapid resource mobilisation	2	(22)
	Clinical or Health Care Worker	Limited clinical case management capacity	2	(22)
	Patient & Community	Inadequate risk communications or community engagement	2	(22)
		Poor sanitation and hygiene practices	1	(11)
	Coordination	Inadequate One Health information sharing/ collaboration	1	(11)
		Weak response coordination, including incident management and rapid response team capacity	1	(11)
		Inadequate multisectoral/disciplinary response teams	1	(11)
	Laboratory	Inadequate diagnostic commodities	1	(11)
		Delayed specimen collection	1	(11)
	Data systems	Lack of timely or complete surveillance data	1	(11)
	Others	Inadequate sources of clean drinking water	2	(22)
		Poor infection prevention control measures in health facilities	1	(11)

*Percentage calculated with number of events with reported bottlenecks as the denominator

**Figure 3.**
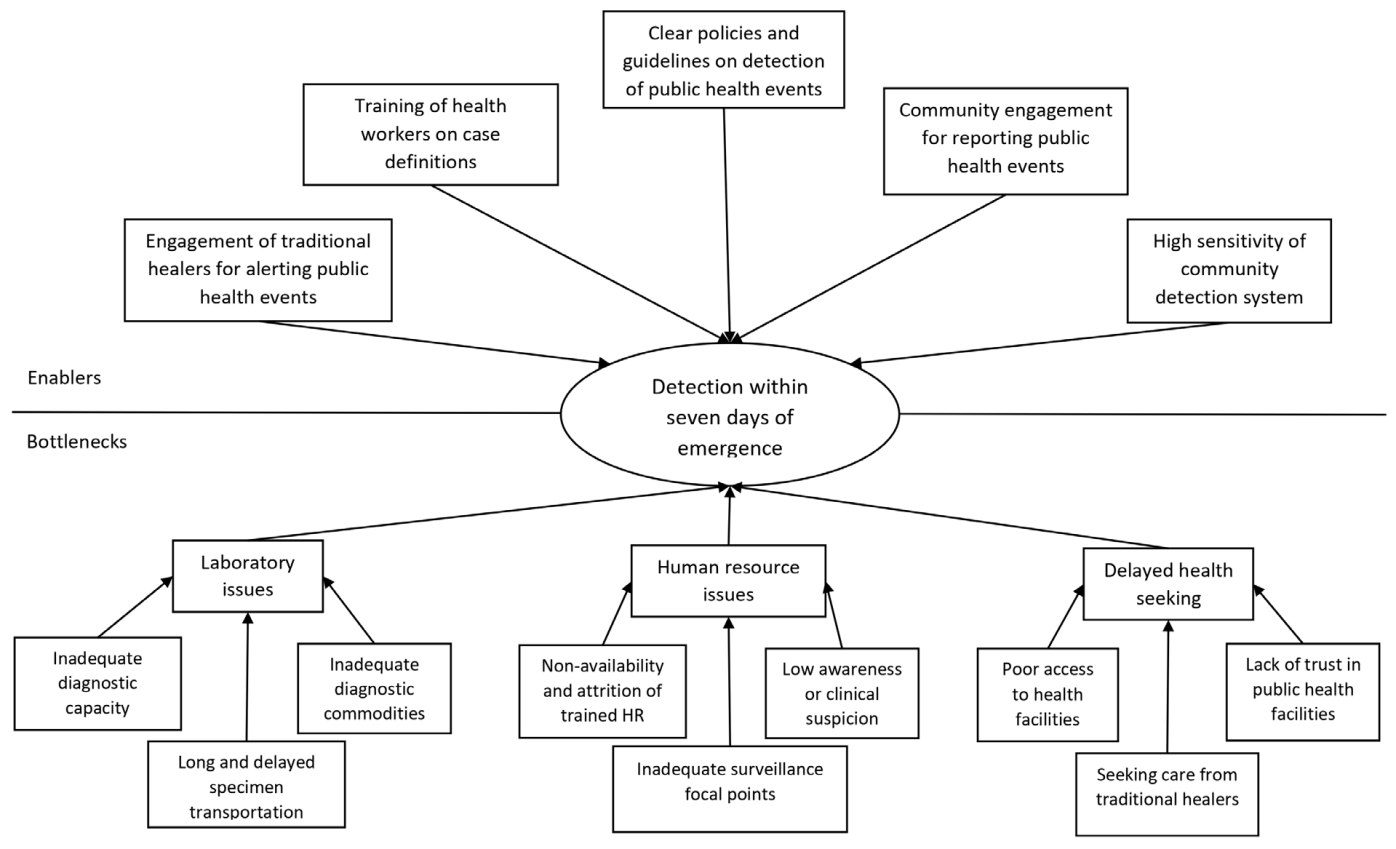
Enablers and bottlenecks for timely detection of a public health event in South Sudan. Figure 3 illustrates the enablers and bottlenecks influencing the timely detection of priority public health events within 7 days of emergence in South Sudan, as identified through in-depth interviews.

Several enablers and bottlenecks in achieving the 7-1-7 target were also brought forth through qualitative interviews with surveillance officers and rapid response teams, which have been included in Figure 3– Figure 5, and Supplementary Table 3, Annex 6, Extended data ^6^. All the perceived bottlenecks matched the ones included in the bottleneck taxonomy created by the 7-1-7 Alliance ^10^.

**Figure 4.**
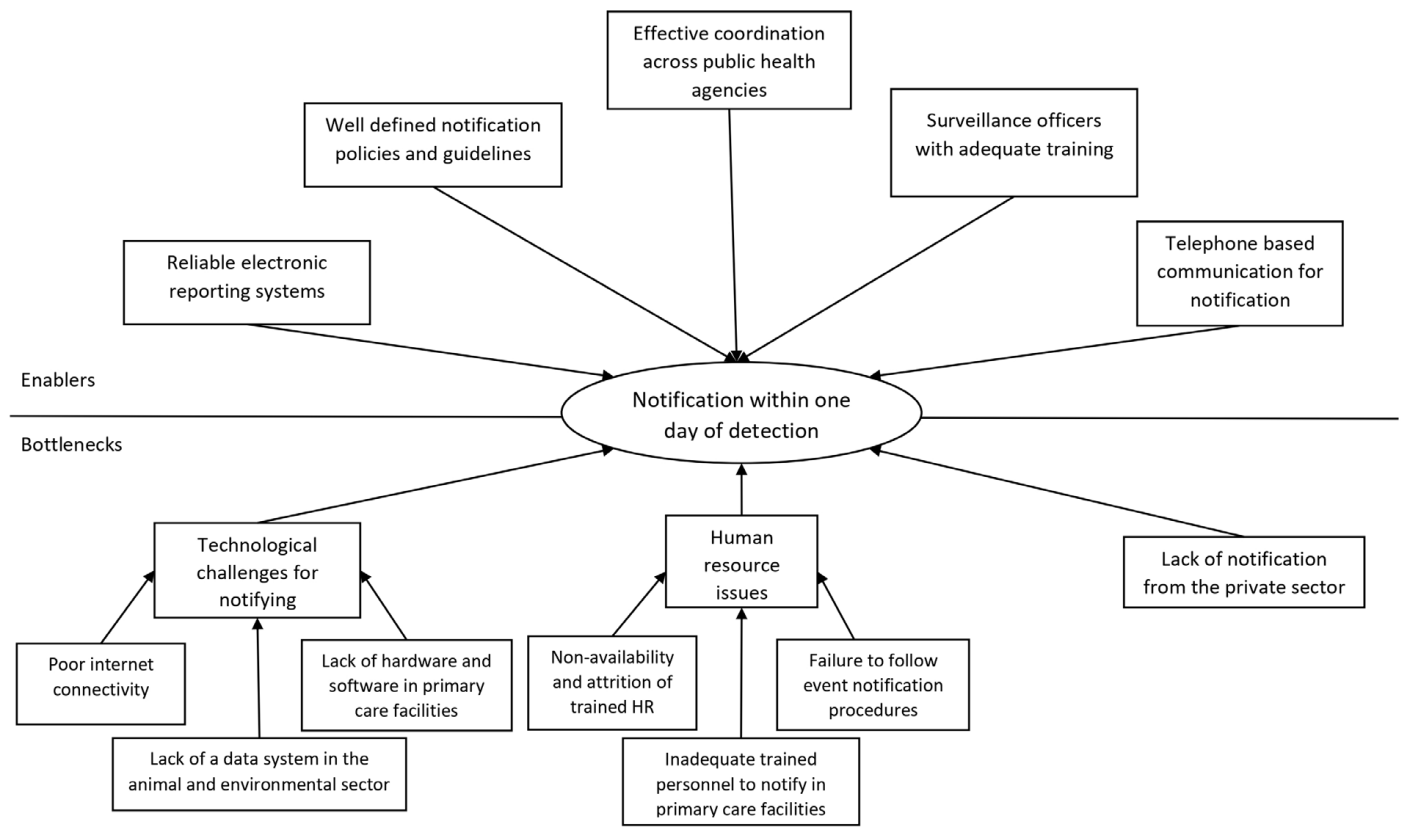
Enablers and bottlenecks for timely notification of a public health in South Sudan. Figure 4 illustrates the enablers and bottlenecks affecting the timely notification of priority public health events within 1 day of detection in South Sudan, as identified through in-depth interviews.

**Figure 5.**
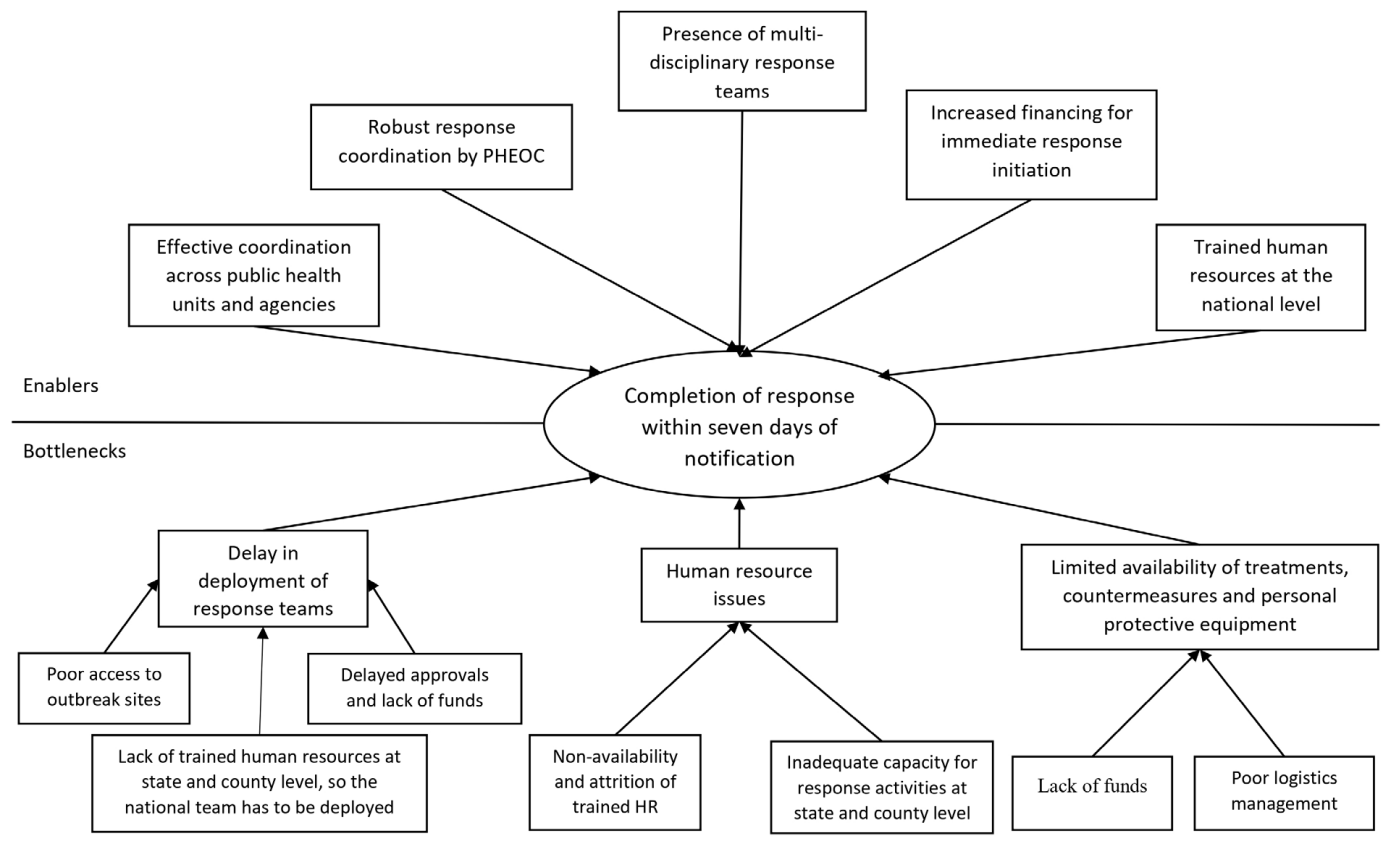
Enablers and bottlenecks for timely response to a public health event in South Sudan. Figure 5 illustrates the enablers and bottlenecks impacting the timely response to priority public health events within 7 days of notification in South Sudan, as identified through in-depth interviews. PHEOC: Public Health Emergency Operations Centre

The national public health emergency call centre, and the community outreach programmes were believed to improve timely detection of events. Additionally, printed resource materials containing surveillance guidelines and case definitions were distributed to surveillance officers at the county, state and national levels to build their capacity on detecting public health events. Training traditional healers and engaging them in alerting events was perceived as a way to overcome delayed detection, since they were often the first point of contact for healthcare. A number of bottlenecks for detecting an event within seven days of emergence were reported, ranging from laboratory issues, human resource issues and delayed healthcare seeking among the population. The poor network and dilapidated condition of roads delayed transportation of both, patients and laboratory specimens. A large number of healthcare posts were vacant because of low government salaries, and lack of clinical suspicion was thought to delay the early diagnosis of certain diseases like mpox.

The enablers for timely notification, as reported by the stakeholders, were the presence of reliable, and functioning electronic reporting systems like EWARS and DHIS-2, well-defined notification guidelines and adequately trained surveillance officers. Phone based notifications also enabled timely notification from areas where there was poor internet connectivity- a key bottleneck for primary care facilities. Additionally, gaps were reported in the animal and environmental sectors. Despite the presence of reporting guidelines, the lack of a functioning reporting system and filled veterinarian posts resulted in poor notification of health events among animals.

Completion of early response actions within seven days of notification was believed to occur because of a number of enablers, including effective coordination. At the national level, the PHEOC enabled coordination across different levels of the health system. Additionally, the One Health Multi-Sectoral Coordination Mechanism enabled coordination between all the One Health line ministries, including Ministry of Environment, Ministry of Animal Resources and Ministry of Health. However, multiple bottlenecks were present which delayed timely response. It was reported that there were no designated government funds for outbreak investigations, and public expenditure on healthcare was also very low. At the subnational level, there were limited availability of functioning RRTs because of the absence of a designated workplace, a lack of resources and vacant posts due to low salaries. Funding of outbreak responses sometimes took two weeks to get approved.

### Objective 3: Challenges in adopting 7-1-7 metrics in humanitarian setting

**Figure 6.**
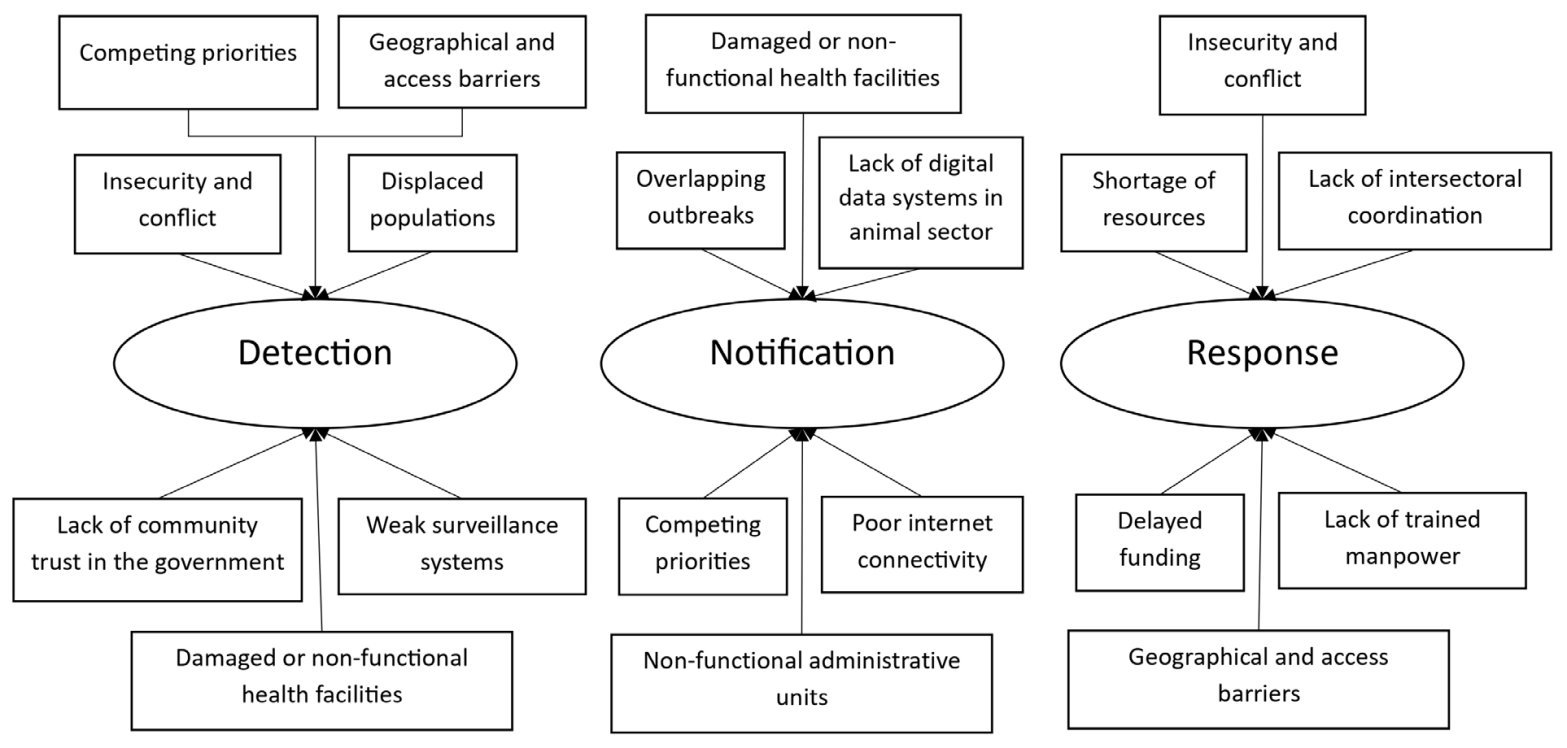
Thematic diagram for challenges in implementing 7-1-7 in humanitarian settings of South Sudan. Figure 6 illustrates the factors challenging the implementation of the 7-1-7 metrics in humanitarian settings in South Sudan, as identified through in-depth interviews.

Through in-depth interviews, we explored challenges in implementing the 7-1-7 framework in humanitarian settings of South Sudan ( Figure 6 and Supplementary Table 4, Annex 6, Extended data) ^6^. Timely detection was hindered by a number of factors, including weak surveillance coverage. Because of insecurity, displacement of populations, empty villages, damaged roads and flooding, surveillance officers often faced difficulty in accessing remote populations. For notifications, along with the lack of internet connectivity, major challenges faced in these resource limited settings were competing priorities and overlapping emergencies. Cases of hepatitis and cholera were detected simultaneously, and health workers prioritised patient care rather than notification. Timely response was challenging because of difficulty in accessing some settlements which had no roads and a boat would have to be used by RRTs, which was not safe. Additionally, RRTs for settlements were previously in place, but due to healthcare staff attrition, they ceased to exist, so the national RRT would be deployed in outbreaks.

## Discussion

This is the first study from South Sudan to comprehensively assess the adoption and use of the 7-1-7 framework, including challenges in humanitarian settings. The study has five key findings. First, South Sudan successfully adopted 7-1-7 by meeting all the adoption milestones at the national level and in four states, facilitated by strong leadership, funding, and technical support from the RTSL. Second, after the adoption of 7-1-7, data availability for applying the 7-1-7 metrics for outbreaks improved. Third, about half of the outbreaks achieved the 7-1-7 target, with delayed health seeking, poor internet connectivity, and inadequate resources being the major bottlenecks for achieving detection, notification, and response targets, respectively. Fourth, the enablers and bottlenecks explored through the qualitative approach mirrored the 7-1-7 bottleneck taxonomy. Finally, non-functional health facilities, weak surveillance systems, competing health emergencies, insecurity, and conflicts hindered adoption in humanitarian settings (including conflict areas, refugee, and internally displaced person camps, and flooded areas).

These study findings provide evidence from South Sudan on the adoption and use of the 7-1-7 metrics in response to the WHO’s Health Security Preparedness Division's call ^4^. Moreover, the challenges identified for adoption, especially in humanitarian settings, would guide countries with similar crises to effectively implement the 7-1-7 metrics in their countries by mitigating the potential threats.

The study has a few strengths. First, the mixed-methods design enabled a comprehensive assessment of the adoption and use of the 7-1-7 metrics in South Sudan. Second, as the study was conducted within routine programmatic settings, the findings reflect the ground realities of the implementation of the 7-1-7 metrics. Finally, we adhered to STROBE and COREQ guidelines for the conduct and reporting of the quantitative and qualitative components, respectively ^11, 12^. The study also had a few limitations. First, the overall sample size of outbreaks for assessing achievements against the 7-1-7 metrics was low, with incomplete data for outbreaks that happened before and after the adoption of the metrics. Second, though data saturation was achieved, the sample size of the qualitative interviews for each objective was relatively small. Finally, as the study was conducted early in the implementation of the 7-1-7 metrics in the country, challenges with the sustainability of applying the 7-1-7 metrics could not be explored. Despite these limitations, the study findings have implications for 7-1-7 implementation for public health emergency preparedness in South Sudan and globally.

First, the successful adoption of the 7-1-7 framework at the national level in South Sudan highlights that even resource-constrained countries can adopt the 7-1-7 metrics with funding and technical support from the 7-1-7 Alliance, leveraging the existing surveillance structures. This successful adoption mirrors the 7-1-7 adoption in neighbouring Uganda and other African countries ^2, 13^. However, South Sudan’s implementation team made some strategic decisions to galvanize political commitment and leadership for the adoption of the 7-1-7 metrics. First, South Sudan instituted separate political and technical 7-1-7 champions instead of just one 7-1-7 champion as done in other countries. Second, tailored 7-1-7 training was delivered to high-level leadership, One Health members, and rapid response teams to achieve political commitment, multi-sectoral engagement, and prepare the frontline team for applying the 7-1-7 metrics, respectively. Third, the team, including the champions, visited Uganda for peer-learning the process of adoption and use of the 7-1-7 metrics. Finally, dissemination of the success story on how applying the 7-1-7 framework enabled policymakers to accelerate the procurement and delivery of yellow fever vaccines during an outbreak in December 2024 increased the buy-in for country-wide adoption of the metrics. Globally, countries aiming to adopt the 7-1-7 metrics should assess the readiness of their surveillance structures, procure financial support for adoption from development partners, avail technical support from the 7-1-7 Alliance, learn from the challenges faced by front-runner countries, and implement contextually appropriate strategies, as seen in South Sudan.

Although national adoption was successful, expanding to all states and counties was not feasible due to the lack of financial resources for training and supporting 7-1-7 teams at the state level. Even in the four states where the 7-1-7 framework was adopted, state teams remained largely non-functional due to insufficient funding and challenges in retaining a trained workforce. Currently, even when the 7-1-7 framework has to be applied to outbreaks occurring in the states where 7-1-7 is adopted, national surveillance officers and personnel from the national RRT are deployed, as the states have no funds. Thus, to achieve functional 7-1-7 adoption in all states of South Sudan, there is an urgent need for sustained funding, either through earmarked funds for 7-1-7 in the health budget or through long-term support from development partners. Similarly, other countries adopting 7-1-7 must also consider financial sustainability and integrate 7-1-7 activities into the routine work process of surveillance teams.

Second, after the adoption of 7-1-7 in South Sudan, there was a clear improvement in the proportion of verified outbreaks to which the timeliness metrics could be applied (36% to 100%), because of the increased availability of data on the date of emergence, detection, notification, or response completion. This was possible due to the training given to surveillance officers and RRTs on the 7-1-7 metrics and the importance of documenting key dates during outbreak investigations and inputting them into the 7-1-7 consolidated data sheet. A similar collateral benefit of instituting the 7-1-7 metrics in improving the overall data quality of public health surveillance systems has been observed in other countries ^13, 14^. However, qualitative exploration revealed that capturing key dates remains a challenge in the animal and environmental sector due to a lack of trained workforce for disease surveillance and notification, and non-existent electronic surveillance recording and reporting systems. Similar challenges with surveillance systems in the animal and environmental sectors have been reported in other African countries ^15^. This threatens the use of the 7-1-7 metrics among the animal and environmental sectors, despite their involvement in 7-1-7 adoption as part of the One Health approach. Thus, there is an urgent need to either institute a relevant surveillance system or to expand EWARS to the animal and environmental sector, and to designate staff responsible for surveillance and notification.

Third, after the adoption of the 7-1-7 metrics, nearly half of the outbreaks in South Sudan met all the targets, with 67% detected within 7 days, 88% notified within 1 day, and 57% with completed response within 7 days. This achievement is similar to findings from the multi-country study (Brazil, Ethiopia, Nigeria, Uganda, and Liberia), which showed that nearly half of all reviewed outbreaks met the complete 7-1-7 criteria, with up to 54% detected within 7 days, 71% notified within 1 day and only 27% with completed response within 7 days. In South Sudan, delayed health seeking, poor internet connectivity, and inadequate resources emerged as the major bottlenecks for achieving detection, notification, and response targets, respectively.

Delayed health seeking is largely due to lack of trust in public health facilities, poor access due to bad roads, and the preference for traditional healers. To address the delayed health-seeking behaviour, there is an urgent need to increase community campaigns to raise awareness about identifying priority public health events and reporting them through the existing hotline number. This would enable the primary healthcare setting to reach out to the community and detect potential public health events swiftly. Similarly, traditional healers across the country must be engaged to identify and report public health events. Also, in the long term, there is a need to improve access to primary healthcare facilities and the quality of care they provide. To overcome the issue of poor internet connectivity for notifying outbreaks, primary care facilities can be equipped with satellite-based internet services that provide high-speed, low-latency internet in remote and rural areas. Human and financial resources have to be earmarked and kept ready for deployment without any bureaucratic delays in initiating response activities.

Fourth, the enablers and bottlenecks for achieving the 7-1-7 metrics from the qualitative exploration closely mirrored the global 7-1-7 bottleneck taxonomy developed by the 7-1-7 Alliance. This confirms the framework’s broad applicability and relevance even in a complex public health system such as South Sudan. However, there is a need for future studies to periodically explore the enablers and bottlenecks using qualitative methods to update the 7-1-7 taxonomy. Additionally, it is necessary to record the ‘action taken report’ for each bottleneck identified while using the 7-1-7 metrics. This would close the loop of feedback and response for each bottleneck identified.

Finally, there are several systemic challenges in expanding the 7-1-7 metrics to humanitarian settings, like conflict-affected areas, refugee and internally displaced person camps, and flooded areas in South Sudan. The non-functional healthcare facilities and weak surveillance systems delay detection, notification, and response. Insecurity and conflict disrupt access to affected populations and interrupt healthcare delivery and surveillance activities. Similar challenges have been reported from other conflict-affected humanitarian settings, where health system disruptions due to violence and population displacement hinder effective disease surveillance and outbreak responses ^16^. To overcome these barriers, it is necessary to partner with NGOs operating in humanitarian settings and build their capacity for instituting effective surveillance structures, including operationalizing EWARS and integrating the 7-1-7 metrics. In line with guidance on adopting 7-1-7, there is a need to develop adaptable models that accommodate access constraints, leverage community-based surveillance, and address funding challenges ^17^.

## Conclusion

This study on the adoption and use of the 7-1-7 metrics in South Sudan for timely outbreak detection, notification, and response showed that it is feasible to adopt the 7-1-7 metrics in a low-income country with humanitarian crises. Strong in-country leadership, funding, and technical support from the 7-1-7 Alliance were the key factors that helped the adoption of 7-1-7 at the national level and in four of the country’s ten states and three administrative areas. Following the adoption of the 7-1-7 metrics in August 2023, the overall quality of data on outbreak detection and response improved in the country, and half of the outbreaks achieved the 7-1-7 target. Given the utility of the 7-1-7 metrics, there is an urgent need for improved funding to expand its adoption to all the states of South Sudan, and to overcome the bottlenecks that delay outbreak detection, notification, and response.

## Data Availability

Figshare: STROBE and COREQ checklists for ‘Adoption and use of the 7-1-7 target for timely outbreak detection, notification and early response in the human and animal sectors in humanitarian crisis-affected South Sudan: A mixed-methods study during 2025’. https://doi.org/10.6084/m9.figshare.30287995 ^9^. Data are available under the terms of the Creative Commons Zero “No rights reserved” data waiver (CC0 1.0 Public domain dedication). Figshare: Data regarding adoption and use of 7-1-7 in South Sudan, https://doi.org/10.6084/m9.figshare.30288880 (Lako 2025) ^18^. This project contains the following underlying data: Checklist for adoption of 7-1-7 in South Sudan.docx (checklist responses for adoption of 7-1-7 at national and subnational level in South Sudan) Key dates, bottlenecks and enablers in South Sudan.xlsx (dates of detection, notification and response of outbreaks from 2013–2025 in South Sudan, along with bottlenecks and enablers) Data are available under the terms of the Creative Commons Attribution 4.0 International license (CC-BY 4.0). Figshare: Annexes for ‘Adoption and use of the 7-1-7 target for timely outbreak detection, notification and early response in the human and animal sectors in humanitarian crisis-affected South Sudan: A mixed-methods study during 2025’. https://doi.org/10.6084/m9.figshare.30290233. (Lako 2025) ^6^. The following extended data supports this project: Extended data_Annexes.docx (Includes the annexes as mentioned in the manuscript, of the data collection tools used, and the supplementary tables) Data are available under the terms of the Creative Commons Attribution 4.0 International license (CC-BY 4.0)
