# Supplementary material for: Adoption and use of the 7-1-7 target for timely outbreak detection, notification and early response in the human and animal sectors in humanitarian crisis-affected South Sudan: A mixed-methods study during 2025
Source: Wellcome Open Res. 2026 Feb 16;11:113. [Version 1] doi: 10.12688/wellcomeopenres.25112.1 (PMC13090763; doi:10.12688/wellcomeopenres.25112.1)
Supplement: Peer review history

# Reviewer report: Reviewer response for version 1

**DOI:** 10.21956/wellcomeopenres.27685.r152533

**Published:** 2026-05-20

**Authors:** Piero Manfredi

**Affiliations:** 1 University of Pisa, Pisa, Italy

This is a well written paper suggesting conditions under which the 7-1-7 approach can be successfully implemented in a low resource setting also suffering a range of humanitarian crises as well a complicated recent history, as is the case of South -Sudan.

 As i am not at expert in the field i have no specific points.

 However, I have a number of more general points

 -- i believe it would be important to discuss not only the fraction of events where 7-1-7 targets were met but especially conditions (and possible determinants) that could be responsible for large departures from targets given the current context and conditions.

 -- From the manuscript, it remains a bit unclear to me which are the key priorities for future steps aiming at

 (i) improving the functionality of 7-17. I understand that a number of bottlenecks was identified but details could be given in terms of e.g. their extent, ability to solve bottlenecks (over which time scales, with which budget requirements etc.)  

 (ii) extending the geographical coverage of 7-1-7 over the country

 (iii) ensuring its sustainability over time.

 Last, i would believe important to add a more extended comparative discussion of the South-Sudan story compared to other African countries which already adopted 7-1-7 in terms of success/bottlenecks and priorities.

Is the work clearly and accurately presented and does it cite the current literature?

Yes

If applicable, is the statistical analysis and its interpretation appropriate?

Yes

Are all the source data underlying the results available to ensure full reproducibility?

Yes

Is the study design appropriate and is the work technically sound?

Yes

Are the conclusions drawn adequately supported by the results?

Yes

Are sufficient details of methods and analysis provided to allow replication by others?

Yes

Reviewer Expertise:

Mathenatical modeling of infectious diseases

I confirm that I have read this submission and believe that I have an appropriate level of expertise to confirm that it is of an acceptable scientific standard, however I have significant reservations, as outlined above.

# Reviewer report: Reviewer response for version 1

**DOI:** 10.21956/wellcomeopenres.27685.r149131

**Published:** 2026-04-17

**Authors:** Ibrahim Franklyn Kamara

**Affiliations:** 1 World Health Organization, Freetown, Sierra Leone

This work is critical as it assesses the implementation of the 7-1-7 approach, which is critical for emergency preparedness and response.

 Abstract

 Since the assessment is on the 7-1-7 approach, kindly include the performance of all the components: detection, notification, and initial early response.

 Method

 You need to justify why the assessment was conducted between 2013 and July 2025. It is not a calendar month approach or a year approach. So kindly state why the data collection was done during this period. Informed consent was verbal or written. The method section should be streamlined to improve readability, if possible 

 Discussion

 The discussion section should also be streamlined to increase readability.

Is the work clearly and accurately presented and does it cite the current literature?

Yes

If applicable, is the statistical analysis and its interpretation appropriate?

Yes

Are all the source data underlying the results available to ensure full reproducibility?

Yes

Is the study design appropriate and is the work technically sound?

Yes

Are the conclusions drawn adequately supported by the results?

Yes

Are sufficient details of methods and analysis provided to allow replication by others?

Yes

Reviewer Expertise:

Emergency preparedness and response, Antimicrobial resistance and stewardship, infection prevention and control, and RMNCAH

I confirm that I have read this submission and believe that I have an appropriate level of expertise to confirm that it is of an acceptable scientific standard.

# Reviewer report: Reviewer response for version 1

**DOI:** 10.21956/wellcomeopenres.27685.r148185

**Published:** 2026-02-28

**Authors:** Palanivel Chinnakali

**Affiliations:** 1 Medical Education and Research,, Puducherry, India

The study looked at the adoption of 7-1-7 framework for outbreak response. Authors have compared the indicators across two time periods and also employed a mixed  methods approach.

 Entire manuscript is lengthy . Authors may consider reducing the word count in Background section. There is a lot of repetitive information in the methods section. Study population for all the objectives can be written in a single paragraph.

 Discussion section is well written. Figures summarizes the key findings of qualitative research findings

Is the work clearly and accurately presented and does it cite the current literature?

Yes

If applicable, is the statistical analysis and its interpretation appropriate?

Yes

Are all the source data underlying the results available to ensure full reproducibility?

Yes

Is the study design appropriate and is the work technically sound?

Yes

Are the conclusions drawn adequately supported by the results?

Yes

Are sufficient details of methods and analysis provided to allow replication by others?

Yes

Reviewer Expertise:

Tuberculosis, Operational Research

I confirm that I have read this submission and believe that I have an appropriate level of expertise to confirm that it is of an acceptable scientific standard.
